# Fluorescence guided surgery imaging systems for breast cancer identification: a systematic review

**DOI:** 10.1117/1.JBO.29.3.030901

**Published:** 2024-03-04

**Authors:** Martha S. Kedrzycki, Hazel T. W. Chon, Maria Leiloglou, Vadzim Chalau, Daniel R. Leff, Daniel S. Elson

**Affiliations:** a Institute of Global Health Innovation, Imperial College London, Hamlyn Centre, London, United Kingdom; b Imperial College London, Department of Surgery and Cancer, London, United Kingdom; c Imperial College Healthcare NHS Trust, Department of Breast Surgery, London, United Kingdom

**Keywords:** fluorescence guided surgery, near-infrared, fluorescent cameras, optical filters, breast conserving surgery

## Abstract

**Significance:**

Breast-conserving surgery (BCS) is limited by high rates of positive margins and re-operative interventions. Fluorescence-guided surgery seeks to detect the entire lesion in real time, thus guiding the surgeons to remove all the tumor at the index procedure.

**Aim:**

Our aim was to identify the optimal combination of a camera system and fluorophore for fluorescence-guided BCS.

**Approach:**

A systematic review of medical databases using the terms “fluorescence,” “breast cancer,” “surgery,” and “fluorescence imaging” was performed. Cameras were compared using the ratio between the fluorescent signal from the tumor compared to background fluorescence, as well as diagnostic accuracy measures, such as sensitivity, specificity, and positive predictive value.

**Results:**

Twenty-one studies identified 14 camera systems using nine different fluorophores. Twelve cameras worked in the infrared spectrum. Ten studies reported on the difference in strength of the fluorescence signal between cancer and normal tissue, with results ranging from 1.72 to 4.7. In addition, nine studies reported on whether any tumor remained in the resection cavity (5.4% to 32.5%). To date, only three studies used the fluorescent signal for guidance during real BCS. Diagnostic accuracy ranged from 63% to 98% sensitivity, 32% to 97% specificity, and 75% to 100% positive predictive value.

**Conclusion:**

In this systematic review, all the studies reported a clinically significant difference in signal between the tumor and normal tissue using various camera/fluorophore combinations. However, given the heterogeneity in protocols, including camera setup, fluorophore studied, data acquisition, and reporting structure, it was impossible to determine the optimal camera and fluorophore combination for use in BCS. It would be beneficial to develop a standardized reporting structure using similar metrics to provide necessary data for a comparison between camera systems.

## 1 Introduction

Breast cancer affects one in eight women worldwide.^1^ With the emergence of ∼287,850 new cases in the United States in 2022,^2^ breast cancer is the most common cancer in women. Approximately 81% of patients receive surgery, either in the form of mastectomy or breast-conserving surgery (BCS). BCS combined with radiotherapy offers comparable oncological outcomes and is preferred in early-stage disease due to improved cosmetic and quality of life outcomes when compared to mastectomy.^3^

During BCS, the tumor is removed *en bloc* with a margin of healthy tissue. However, one of the unresolved challenges during BCS is the risk of positive resection margins (PMR), whereby the tumor extends up to the edge of the removed specimen.^4^ PMR implies a risk of residual tumor in the resection bed following excision, which significantly increases the risk of ipsilateral recurrence.^4^ Therefore, in order to mitigate this risk, women with positive margins typically undergo re-operation.^5^^–^^8^ On average, one in five women (ranging from 10% to 60%) undergoes re-operation after failed index BCS.^9^ Approaches to tackle high rates of re-operative intervention include tumor localization and identification techniques.

**Fig. 1:**
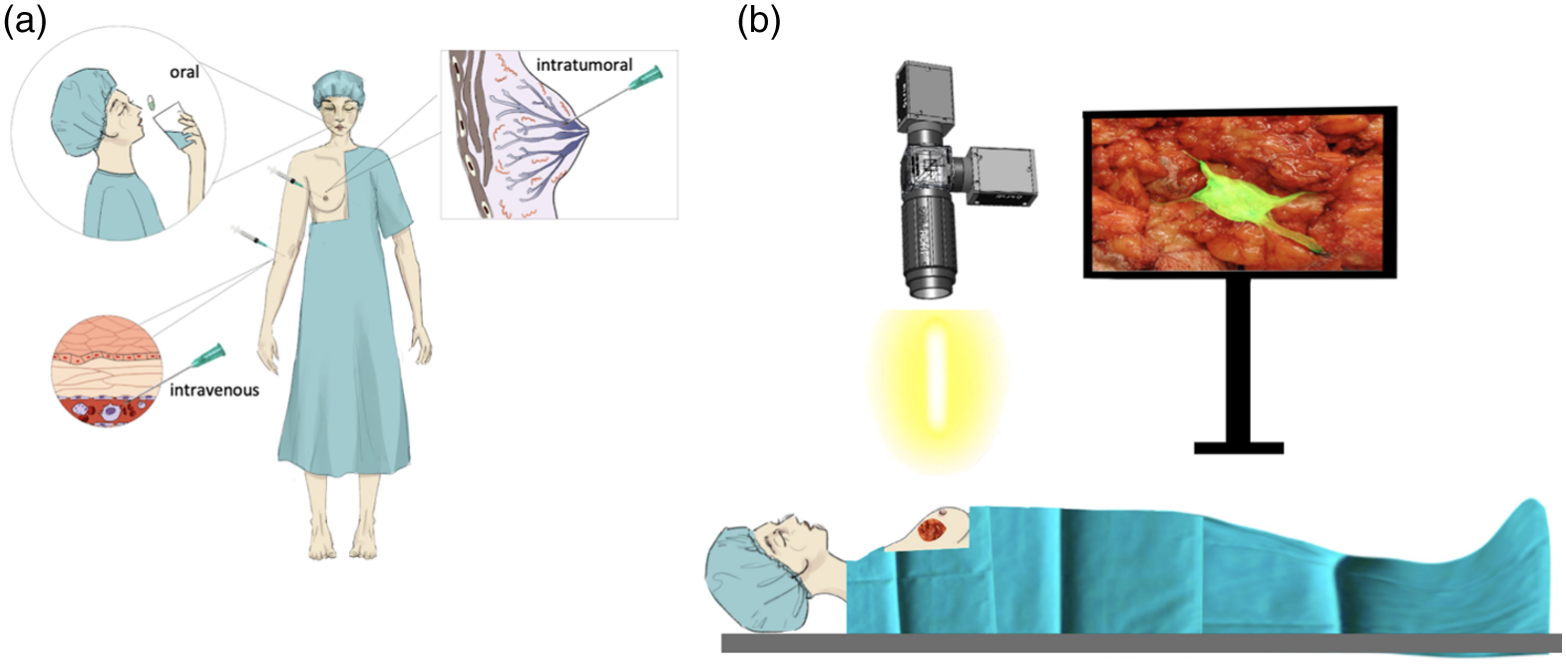
FGS in breast cancer. (a) The patient is administered a fluorescent agent either via an oral solution or an injection (either into the tumor or into the systemic circulation). This fluorophore then targets the tumor actively (i.e., by targeting receptors or enzymes) or passively (i.e., by leaking into the tumor). (b) A light source emits a specific range of wavelengths of light to excite that agent. Images of the operative area are acquired using a camera sensitive to fluorescence. These images are taken of the tumor *in situ*, with the surgeon’s view of the operating field undisturbed. The image displayed on the top right screen is the fluorescence camera processed image wherein the likely site of the tumor (green) is superimposed onto the color image. A visual depiction of the areas of fluorescence is available to the operating surgeon for improved intraoperative decision making.

Several techniques are available for pre-operative tumor localization, including wire-guided localization, radio-guided occult lesion localization, or seed guidance [e.g., radioactive seeds or Magseed® (Endomag, Cambridge, United Kingdom)].^10^ Although certain reports have demonstrated a reduction of 10% to 30% in positive margins when compared to palpation guided surgery, they only provide approximate guidance for localization of the center of the tumor.^6^^,^^10^^–^^12^ The necessity for innovative techniques to revolutionize localization and reduce re-operation rates has led to the development of fluorescence-guided surgery (FGS), a technique that utilizes specialist imaging systems in combination with fluorescent probes to visualize malignant tissue intraoperatively (Fig. 1).^13^ Fluorescent probes accumulate in malignant tissue either by targeting receptors, targeting enzymes, or by passively leaking into the tumor.

**Fig. 2:**
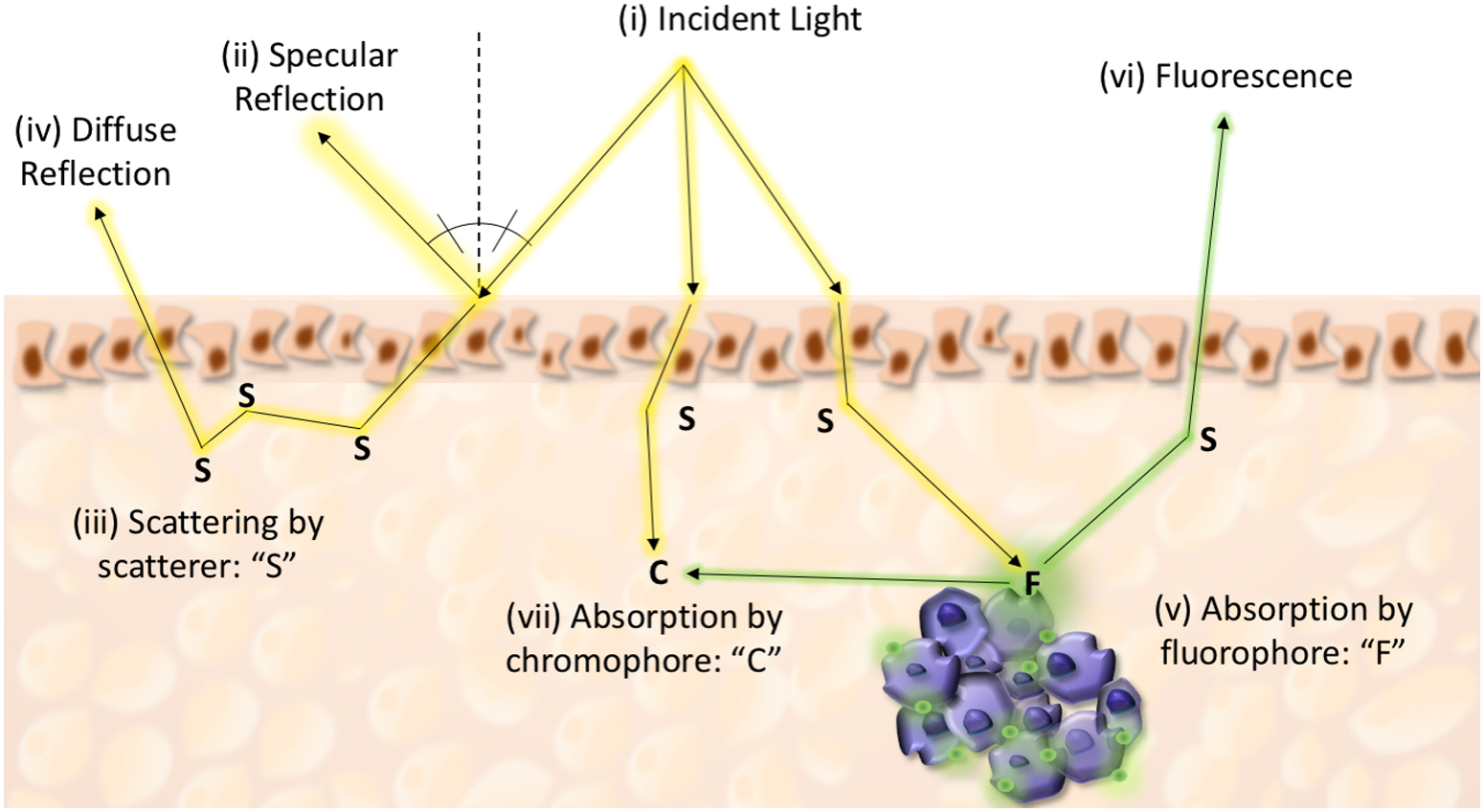
Properties of light in tissue. Illustration of light–tissue interactions. Upon illumination of the tissue, part of the incident light (i) is reflected from the tissue surface without changing its initial properties (spectral shape or polarization state). This reflection is called “specular reflection” (ii), whereby both incident and reflected light are coplanar and at the same angle to the surface normal (perpendicular to the surface direction). Part of incident light can also be scattered (iii) in the tissue and re-emerge from the surface. This light is called “diffuse reflection” (iv) and its direction/spectral shape and polarization state are altered compared to the incident light. Finally, part of the incident light can be absorbed by a fluorophore (v), whereby part of the initial energy will be emitted as fluorescence (vi), or absorbed by a chromophore (viii), whereby no subsequent fluorescence emission occurs. Fluorescence light can be absorbed or scattered as well prior to its emergence from surface. Image reproduced with the permission of publisher.^18^

Fluorescence imaging uses three fundamental hardware components: a light source, a digital camera, and optical filters to limit the spectral band emitted by the light source detectable by the camera to ensure efficient excitation and detection of fluorescence.^14^ A major benefit of FGS is that it works in real-time and does not expose patients to ionizing radiation. In FGS, the majority of cameras work in the near-infrared spectral range (wavelengths from 780 to 1000 nm) as this enables significant contrast from tissue autofluorescence (wavelengths 400 to 780 nm).^15^ As this allows optical penetration of up to 4 mm,^16^ it fulfils the guidelines set out by the Society of Surgical Oncology and American Society for Radiation Oncology (i.e., no tumor at inked margin for invasive breast cancer and 2 mm for ductal carcinoma *in-situ*).^17^ However, the main impediment of FGS is absorption and scattering of the light by other tissue components, as is shown in Fig. 2.

Commercially available camera systems, such as the Photodynamic Eye™ (Hamamatsu Photonics, Shizuoka, Japan), Fluobeam 800™ (Fluoptics, Grenoble, France), and SPY™ (Novadaq Technologies, Toronto, Canada), have been increasingly used in clinical studies.^5^ In addition, various custom-built FGS imaging systems are currently under development for use in breast cancer surgery.

While there have been multiple clinical trials using various camera systems toward improving precision in breast cancer surgery,^19^^–^^25^ there are no reviews comparing systems to investigate efficacy or diagnostic accuracy. Therefore, our aim was to systematically review the current evidence on FGS imaging systems for intraoperative breast cancer diagnosis.

## 2 Methods

### 2.1 Ethics

This systematic review was conducted according to Preferred Reporting Items for Systematic Reviews and Meta-Analysis guidelines. The study was registered on PROSPERO (CRD42021286487). No ethical approval was required.

### 2.2 Strategy for Identification and Selection of Studies

Embase, MEDLINE, Web of science, and Scopus were systematically searched for all articles published before December 2022. The search was conducted using the following Medical Subject Headings (MeSH): “fluorescence” AND “breast cancer” AND “surgery” AND “fluorescence imaging.” For different databases, the search terms were adjusted as required. Additional reports were identified using Google Scholar and the CLEARER database (of FGS used in cancers) through citation tracking. The full search strategy can be found in the Appendix.

Covidence systematic review software (Veritas Health Innovation, Melbourne, VIC, Australia)^26^ was used for duplicate removal, title and abstract screening, full-text review, and data extraction.

### 2.3 Eligibility Criteria

Studies were included in this review if: (1) a fluorescence camera system was used to assess breast cancer and/or surgical cavities; (2) contrast agents were utilized; and (3) the full text was available in the English language. Studies were excluded if: (1) the optical imaging system was for pre-operative cancer diagnosis; (2) spectroscopy (but not imaging) was used; (3) the studies included only benign breast tissue lesions, only sentinel lymph nodes, or non-breast cancers; or (4) the report was a review, case report, poster, abstract, project proposal, expert opinion, animal study, or cell line study.

### 2.4 Study Selection and Data Synthesis

Data were screened and extracted by two authors independently, MK and HC. Disagreements were resolved with the senior authors (DRL and DE). Sociodemographic variables including sample size, age, and body mass index (BMI) were collected. With regards to cancer characteristics, genotype (e.g., invasive ductal carcinoma), immunophenotype (ER, PR, HER2), and use of pretreatment (i.e., neoadjuvant chemotherapy or hormonal therapy) were determined. Elements describing the imaging systems themselves and the contrast agents they were paired with (including dosage, route of administration, excitation, and emission wavelengths) were identified. Lastly, outcomes such as tumor to background ratio (TBR), positive margin assessments, diagnostic accuracy (including sensitivity, specificity, positive predicted value), re-excision rate, and any adverse events were recorded.

## 3 Results

### 3.1 Search and Selection of Articles

1182 articles were identified, of which, 372 studies were removed after de-duplication. This resulted in 810 studies undergoing title and abstract screening, of which, 692 studies failed to meet the inclusion criteria. An additional 40 studies were identified through bibliographic cross-referencing, and out of the 157 reports that were assessed in detail for eligibility, only 21 studies met all criteria for inclusion in the review.

### 3.2 Patient Demographics

Overall, there were 12 prospective clinical trials.^14^^,^^19^^–^^23^^,^^25^^,^^27^^–^^32^ The nine remaining studies were either case series, feasibility trials, or cohort studies.^24^^,^^33^^–^^39^ Overall, these studies encompassed 894 patients receiving optical imaging in conjunction with contrast agents in breast cancer tissue assessment. Table 1 summarizes patient demographics and cancer subtypes studied.

**Table 1:** Summary of study type, patient demographics, and cancer clinicopathological data.

Author/year	Registry numbers	Method	No. of patients	Mean age in years (range)	Mean BMI in $\mathrm{kg}/m^{2}$ (range)	Tumor subtype	Immunophenotype
Dintzis et al. ^19^	NCT02496065	NRCT	23	NA	NA	DCIS (5)	ER+, HER2− (19)
						IDC + DCIS (18)	ER−, HER2+. (0)
						ILC + LCIS (3)	Triple+ (1)
						MUC + DCIS (1)	Triple− (2)
Keating et al. ^27^	University of Pennsylvania Institutional Review Board	NRCT	12	60^b^ (44–70)	NA	IDC (9), ILC (3)	ER+, HER2− (7)
							ER−, HER2+ (0)
							Triple+. (2)
							Triple− (1)
Kedrzycki et al. ^14^ ^d^	REC 19/LO/0927	NRCT	40^a^	51.2 (33–81)	26.1 (19–36.6)	DCIS (4), IDC (6)	ER+, HER2− (31)
						IDC + DCIS (23)	ER−, HER2+. (1)
						ILC + LCIS (4)	Triple+ (2)
						MUC + DCIS (1)	Triple- (1)
						IMPC (1)	
Koch et al. ^20^	NCT01508572	NRCT	19	64.6	NA	NA	ER+, HER2− (n)
							ER−, HER2+ (n)
							Triple+ (n)
							Triple− (n)
Koller et al. ^21^	NCT02583568	NRCT	26	63.3 (49–77)	NA	ICNST (19)	^c^
						PC (1)	
Lamberts et al. ^40^	NCT01508572	Feasibility	20	65^b^ (46–81)	NA	IDC (17)	^c^
						IDC + ILC (3)	
Lee et al. ^34^	NCC2016-0071	Case control	414	54.1	NA	DCIS (50)	^c^
						IDC (364)	
Leiloglou et al. ^33^	REC 18/LO/2018	Feasibility	10^a^	56 (15–70)	24.2 (19.2–30.2)	IDC (3)	ER+, HER2− (n)
						IDC + DCIS (5)	ER−, HER2+ (n)
						ILC + DCIS (1)	Triple+ (n)
						MUC (1)	Triple− (n)
Leiloglou et al. ^28^ ^d^	REC 19/LO/0927	NRCT	40^a^	51 (33–81)	26.1 (19–36.6)	DCIS (4), IDC (6)	ER+, HER2− (31)
						IDC + DCIS (23)	ER−, HER2+ (1)
						ILC + LCIS (4)	Triple+ (2)
						MUC + DCIS (1)	Triple− (1)
						IMPC (1)	
Liu et al. ^35^	Institutional Ethics Committee of Dalian Central Hospital	Cohort	56	53.8^b^ (34–78)	NA	DCIS (6)	ER+, HER2− (n)
						IDC (50)	ER−, HER2+ (n)
							Triple+ (n)
							Triple− (n)
Ottolino-Perry et al. ^22^	NCT01837225	RCT	45	55.6	NA	IDC (37)	^c^
						ILC (6)	
Park et al. ^36^	NA	Cohort	10	NA (55–75)	NA	NA	ER+, HER2− (n)
							ER−, HER2+ (n)
							Triple+ (n)
							Triple− (n)
Pop et al. ^41^	NCT02027818	Cohort	35 (50 recruited but 15 excluded)	63 (27–79)	NA	IDC (32)	NA
						ILC (3)	
Smith et al. ^23^	Massachusetts General Hospital Institutional Review Board	NRCT	15	63^b^ (48–78)	NA	DCIS (4)	ER+, HER2− (11)
						IDC + DCIS (8)	ER−, HER2+ (0)
						ILC (1)	Triple+ (1)
						IDC + ILC + DCIS (1)	Triple- (0)
						IDC + ILC + DCIS (1)	
Smith et al. ^24^	NCT03321929	Feasibility	45	59^b^ (44–79)	27.6 (20.4–44.4)	IDC +/− DCIS (25)	ER+, HER2− (n)
						ILC (5)	ER−, HER2+ (n)
						IDC + ILC + DCIS (3)	Triple+ (n)
						DCIS (12)	Triple− (n)
Tong et al. ^30^	Institutional Ethics Committee of Dalian Central Hospital	RCT	32	52.5 (26–79)	NA	NA	^c^
Tummers et al. ^31^	Leiden University Medical Center Medical Ethics Committee	NRCT	24	60 (44–82)	24 (19–37)	IDC (15)	ER+, HER2− (19)
						ILC(4)	ER−, HER2+ (11)
						DCIS (3)	Triple+ (1)
						MUC (1)	Triple− (2)
						PMC (1)	
Tummers et al. ^38^	Leiden University Medical Center Medical Ethics Committee	Case series	3	NA (53–61)	NA	ICNST(2)	ER+, HER2− (1)
						Metaplastic carcinoma (1)	ER−, HER2+ (0)
							Triple+ (0)
							Triple− (2)
Unkart et al. ^25^	NCT02391194	NRCT	27^a^	NA (32–69)	NA	IDC (16)	ER+, HER2− (24)
						ILC (4)	ER−, HER2+ (3)
						MUC (2)	Triple+ (1)
						DCIS (2)	Triple- (2)
						Mixed (2)	
Veys et al. ^37^	NCT02032563	Cohort	8^a^	52.4 (31–65)	NA	IDC(8)	ER+, HER2− (3)
						ILC (1)	ER−, HER2+ (4)
							Triple+ (0)
							Triple− (2)
Zhang et al.^32^	ChiCTR1800015400	NRCT	30^a^	55 (32–68)	24 (18.8–34)	IDC (12)	ER+, HER2− (14)
						IDC + ILC + MUC (1)	ER−, HER2+ (15)
						IDC + ILC (1)	Triple+ (0)
						DCIS + IDC (2)	Triple- (1)
						DCIS(3)	

BMI, body mass index; NRCT, non-randomized controlled trial; RCT, randomized controlled trial; DCIS, ductal carcinoma *in situ*; IDC, invasive ductal carcinoma; ILC, invasive lobular carcinoma; LCIS, lobular carcinoma *in situ*; MUC, mucinous carcinoma; FAD, fibroadenoma; IMPC, invasive micropapillary carcinoma; ICNST, invasive carcinoma of no specific type; PC, papillary carcinoma; PMC, primary mucoepidermoid carcinoma, ER, estrogen receptor; HER2, human epidermal growth factor receptor.

a Neoadjuvant chemotherapy (NACT) administered.

b Median value is provided instead of mean.

c ER and HER2 status written independently of each other.

d The studies by Leiloglou et al.^33^ and Kedrzycki et al.^14^ used the same camera system and same 40-patient group but with different image processing methods. For the purpose of this paper, data were only counted once.

Four studies described ethnicity,^14^^,^^24^^,^^27^^,^^28^ but only one reported on menopausal status.^23^ Five studies included patients who underwent neoadjuvant chemotherapy (NACT) prior to BCS or mastectomy. Unkart et al.^25^ included five pretreated patients out of 27, Veys et al.^37^ encompassed eight pretreated patients, and Kedrzycki et al.^14^ and Leiloglou et al.^28^ reported 2 out of 40 patients that had received NACT. However, only Zhang et al.^32^ investigated the impact of patients with NACT compared to those with primary surgery using a custom-built camera system. They reported a significant difference (p<0.05) in fluorescence detection rate and strength of signal, whereby only 30% of NACT cases were detected with a TBR of 1.63 in contrast to 80% of primary cases with a TBR of 1.94.^32^

### 3.3 Imaging Systems

Table 2 summarizes the imaging systems and their diagnostic accuracy. A total of 11 different imaging systems were reported. Studies using Food and Drug Administration approved camera systems permitted for purposes other than breast cancer included: two studies which exploited the Photodynamic Eye™ (PDE) camera system (Hamamatsu Photonics, Shizuoka, Japan),^24^^,^^30^ two employed Fluobeam 800™ imaging system (Fluoptics, Grenoble, France),^37^^,^^41^ two utilized the Artemis™ fluorescence imaging system (Quest Medical Imaging, Middenmeer, The Netherlands),^27^^,^^38^ two capitalized on the mini-FLARE^TM^ (Beth Israel Deaconess Medical Center, Boston, Massachusetts),^31^^,^^36^ and one study used the Visual Navigator^TM^ camera system (SH System, Gwangju, South Korea).^34^

**Table 2:** Comparison of diagnostic accuracy between different fluorescence imaging systems.

Author/year	Imaging system	Contrast agent	Dose	Route	Time of administration	TBR	TBR calculation	PMR (%)	Sn	Sp	PPV	Site of image acquisition			
												*In-vivo* tumor	*Ex-vivo*	*In-vivo* cavity	Cut-up
Dintzis et al.^19^	SIRIS™	Tozuleristide	6 mg, 12 mg	IV	1 to 26 h preop	NA	NA	NA	NA	NA	NA	**X**	**√**	**X**	**√**
Keating et al.^27^	Artemis™	ICG	5 mg/kg	IV	24 h preop	*In-vivo*: 3.14 ± 0.34	ROI tumor/ROI bkgd	NA	NA	NA	NA	**√**	**√**	**√**	**X**
						*Ex-vivo*: 3.46 ± 0.35									
Kedrzycki et al.^14^	Custom built	ICG	0.25 mg/kg	IV	EPR: at anesthetic induction	EPR: 2.10 ± 0.92	Matched tumor/bkgd	32.5	EPR: 0.66	EPR: 0.90	NA	**√**	**√**	**√**	**√**
					Angio: immediately prior to tumor extraction	Angio: 3.18 ± 1.74			Angio: 0.82	Angio: 0.93					
Koch et al.^20^	EagleRay-V3	Bevacizumab–IRDye800cw	4.5 mg	IV	3 days preop	1.8 to 9	ROI tumor/ROI bkgd (relative to pt)	NA	0.98	0.79	NA	**√**	**√**	**X**	**X**
Koller et al.^21^	System by Surgvision	Bevacizumab–IRDye800cw	4.5, 10, 25, 50 mg	IV	3 days preop	1.8 (10 mg)^c^	ROI/bkgd	30	NA	NA	NA	**√**	**√**	**√**	**√**
						3.1 (25 mg)^c^									
Lamberts et al.^40^	EagleRay-V3	Bevacizumab–IRDye800cw	4.5 mg	IV	3 days preop	NA	NA	10	NA	NA	NA	**X**	**X**	**√**	**X**
Lee et al.^34^	Visual navigator	ICG	25 mg	Intralesional^b^	At anesthetic induction	NA	NA	10.5	NA	NA	NA	**√**	**√**	**√**	**X**
Leiloglou et al.^33^	Custom built	ICG	12.5 mg	IV	Angio: immediately prior to tumor extraction	NA	NA	NA	NA	NA	NA	**√**	**√**	**√**	**X**
Leiloglou et al.^28^ ^a^	Custom built	ICG	0.25 mg/kg	IV	EPR: at anesthetic induction	NA	Matched tumor/bkgd (*ex-vivo* and histo)	NA	0.75 +/− 0.3	0.89 +/− 0.2	NA	**√**	**√**	**√**	**√**
					Angio: immediately prior to tumor extraction										
Liu et al.^35^	PDE	ICG	NA	Intralesional^a^	NA	NA	NA	5.4	NA	NA	1.00	**√**	**X**	**√**	**X**
Ottolino-Perry et al.^22^	PRODIGI	5-ALA	15 mg/kg, 30 mg/kg	Oral	2 to 4 h pre-op	NA	ROI tumor/ROI bkgd (of fresh bisected specimen)	15.6	Low dose: 0.65	Low dose: 0.85	Low dose: 0.77	**X**	**√**	**√**	**X**
									High dose: 0.68	High dose: 0.80					
											High dose: 0.75				
Park et al.^36^	Mini-FLARE	PM700-Ca, PM800-SO3	NA	Applied onto excised lesion	Post-op (applied to specimen)	NA	NA	NA	NA	NA	NA	**X**	**√**	**X**	**X**
Pop et al.^41^	Fluobeam 800	ICG	0.25 mg/kg	IV	At anesthetic induction	1.8 ± 0.7	NA	14.7	NA	0.60	0.29	**X**	**√**	**√**	**X**
Smith et al.^23^	LUM	LUM015	0.5 mg/kg, 1.0 mg/kg	IV	NA	0.5 mg/kg: 4.70 ± 1.23	ROI tumor/ROI bkgd (of fresh bisected specimen)	NA	NA	NA	NA	**X**	**X**	**√**	**√**
						1.0 mg/kg: 4.22 ± 0.96									
Smith et al.^24^	LUM	LUM015	1.0 mg/kg	IV	56 to 402 min preop	NA	ROI tumor/ROI bkgd (of fresh bisected specimen)	17.8	0.84	0.73	NA	**X**	**√**	**√**	**X**
Tong et al.^30^	PDE	ICG	10 mg	Intralesional^a^	NA	NA	NA	12.5	NA	NA	NA	**√**	**X**	**√**	**X**
Tummers et al.^31^	Mini-FLARE	MB	1 mg/kg	IV	3 h pre-op	2.40 ± 0.80	ROI tumor/ROI bkgd	17.0	NA	NA	NA	**X**	**√**	**X**	**√**
Tummers et al.^38^	Artemis	EC17	0.1 mg/kg	IV	2 to 3 h pre-op	2.3	NA	NA	NA	NA	NA	**X**	**√**	**X**	**X**
Unkart et al.^25^	System not specified	AVB-620	1, 2, 4,8, 16 mg	IV	2 to 20 h pre-op	Tumor ratio value: 1.09 ± 0.18	ROI tumor/ROI bkgd	NA	NA	NA	NA	**√**	**√**	**√**	**X**
						Adjacent tissue ratio value: 0.59 ± 0.04									
Veys et al.^37^	Fluobeam 800	ICG	0.25 mg/kg	IV	At anesthetic induction	3.3 ± 1.68	ROI tumor/ROI bkgd	NA	0.94	0.32	NA	**X**	**√**	**X**	**X**
Zhang et al.^32^	Custom built	MB	0.5 mg/kg	IV	3 h pre-op	No NACT: 1.94 ± 0.71	ROI tumor/ROI bkgd	NA	0.63	NA	0.79	**X**	**X**	**√**	**X**
						NACT: 1.63 ± 0.38									

TBR, tumor-to-background ratio; bkgd, background; PMR, positive margin resection rate; Sn, sensitivity; Sp, specificity; PPV, positive predictive value; SIRIS, synchronized infrared imaging system; IV, intravenous; hrs, hours; preop, preoperatively; ICG, indocyanine green; ALA, aminolevulinic acid; PDE, photodynamic eye; PRODIGI, portable real-time optical detection identification and guide for intervention; MB, methylene blue; NACT, neo-adjuvant chemotherapy; EPR, enhanced permeability and retention; Angio, angiography.

TBR = mean fluorescence intensity of pixels from region of interest (ROI) in tumor/ mean fluorescence intensity of pixels from the background ROI.

a Also included diagnostic accuracy of 0.84 ± 0.2.

b Intralesional injection employs US guidance to inject a fluorescent contrast medium into the core of the tumor (as is the case when mapping sentinel nodes).

c There are 2 TBRs listed in the study by Koller et al. which are relative to the dose administered. The 10 mg dose of bevacizumab resulted in a TBR of 1.8, and the 25 mg dose in a TBR of 3.1.

The remaining studies included two that deployed the LUM fluorescence imaging system (Lumicell, Inc., Newton, Massachusetts),^23^^,^^24^ one that used the synchronized infrared imaging system (SIRIS) (Teal Light Surgical, Inc., Seattle, Washington),^19^ two that capitalized on the EagleRay-V3 (Technical University of Munich, Munich, Germany),^20^^,^^40^ and finally one that utilized portable real-time optical detection identification and guide for intervention (PRODIGI) handheld fluorescence imaging system (SBI-ALApharma Canada Inc., Toronto, Canada).^22^ Two studies used an unspecified camera system (system by SurgVision, Harde, The Netherlands),^21^ and the remaining studies^14^^,^^25^^,^^28^^,^^32^ that developed in-house camera systems did not include specified model or company name.

### 3.4 Contrast Agents and Tumor-to-Background Ratio

**Fig. 3:**
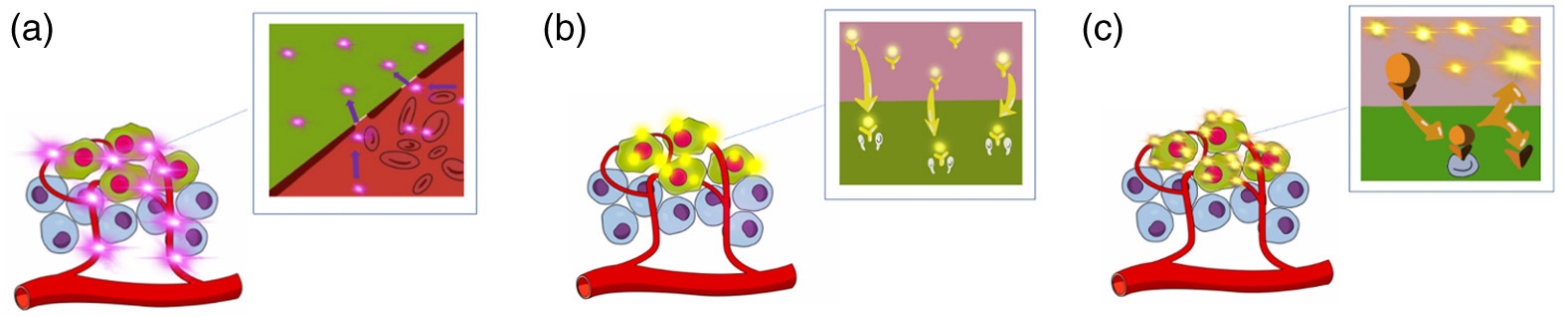
Mechanism of action for targeting tumors using fluorophores. (a) Passive targeting through the EPR effect whereby the fluorophore leaks into tissue due to the porous vasculature and has impaired lymphatic outflow. Panels (b) and (c) illustrate active targeting. (b) The targeting of specific receptors overexpressed in tumor. (c) The targeting of specific enzymes present in the tumor microenvironment by requiring the probe activation by that enzyme.

There were eleven studies which used the nonspecific passive fluorophores indocyanine green (ICG) and methylene blue (MB).^14^^,^^27^^,^^28^^,^^30^^–^^35^^,^^37^ These fluorophores take advantage of the enhanced permeability and retention (EPR) effect, whereby they leak into the tumor via porous vasculature and remain there due to impaired lymphatic outflow [Fig. 3(a)].^14^^,^^27^^,^^28^^,^^30^^–^^35^^,^^37^ Of the nine studies deploying ICG, three used the same custom built camera system^14^^,^^28^^,^^33^ and six used commercially accepted imaging systems.^27^^,^^30^^,^^34^^,^^35^^,^^37^ Six studies encompassing 105 patients, administered 0.25  mg/kg, 5  mg/kg, or 12.5 mg intravenous ICG.^14^^,^^27^^,^^28^^,^^33^^,^^37^ In these studies, TBR varied from 1.72 to 3.46, irrespective of the disparity in ICG doses. Three studies administered 10 or 25 mg ICG intralesionally.^30^^,^^34^^,^^35^ Of the two studies that capitalized on MB,^31^^,^^32^ one study employed the Mini-FLARE^31^ and one utilized a custom-built camera system.^32^ Both studies administrated MB intravenously and reported a similar range of TBR (1.94±0.71 and 2.40±0.80).^31^^,^^32^

Only Kedrzycki et al.^14^ and Leiloglou et al.^28^ assessed the effects of two different timings of administration, one immediately prior to resection and the other at the start of the operation. Kedrzycki et al. observed that ICG administration immediately prior to resection had a statistically significant higher TBR in both *ex-vivo* and histopathology cut-up than the start of the operation (2.10±0.6 and 3.18±1.74 versus 1.72±0.31 and 2.10±0.92 relatively).^14^ However, the differences in sensitivity and specificity were not statistically significant except for certain cases where texture metrics were applied.

One study evaluated the fluorescence of protoporphyrin IX, a metabolite of ALA, whose accumulation is caused by metabolic disruption in the heme formation pathway in breast cancer cells.^22^ The study by performed by Ottolino-Perry et al.^22^ was a phase 1 safety and comparative study of oral 15 and 30  mg/kg ALA utilizing the PRODIGI custom-built camera.^22^ Although they did not report a TBR, they reported a statistically significant difference between patients who had received ALA and control patients (p<0.05).^22^

There were five studies that targeted specific receptors including bevacizumab–IRDye800CW (vascular endothelial growth factor), EC17 (folate), and tozulesteride (chloride channels), all of which were administered intravenously [Fig. 3(b)]. Three studies administered 4.5, 10, 25, and 50 mg bevacizumab–IRDye800CW,^20^^,^^21^^,^^40^ two exploiting the Eagle-Ray custom-built camera system^20^^,^^40^ and one study utilizing a system developed by SurgVision.^21^ Among these studies, only Koch et al.^20^ reported a TBR, ranging from 1.8 to 9.0. Only Lamberts et al.^40^ compared the concentration of bevacizumab–IRDye800CW to VEGF-A levels in tumor versus healthy tissue and found a direct correlation between the two.

One study examined EC17 in combination with the Artemis camera system; however, the authors reported that there was too much background noise from the autofluorescence of normal breast tissue to enable tumor identification.^38^ Lastly, there was a phase two comparative study evaluating 6 and 12 mg of tozulesteride in combination with the SIRIS camera system *in-vivo*; however, TBR was not reported.^19^ Although targeting calcium deposits instead of tumor receptors, one study applied PM700-Ca and PM800-SO3 on excised breast tissue and combined it with the mini-FLARE to evaluate pre-cancerous ductal carcinoma *in-situ*.^36^

Three studies used enzyme targeting fluorophores [Fig. 3(c)].^23^^,^^24^^,^^42^ Two studies administered LUM015,^23^^,^^24^ which requires cleavage by cathepsin to be activated. Utilizing the LUM imaging system, this combination exhibited the highest TBR demonstrating 4.70±1.23 and 4.22±0.96 when receiving a 0.5 and 1.0  mg/kg dose, respectively.^23^ One study by Unkart et al.^25^ capitalized on AVB-620, which requires activation via matrix metalloproteinases. They recorded a TBR of 1.85±11.^25^

Ten studies calculated TBR using the average pixel fluorescence intensity in the entire region of interest in the tumor versus background on the *ex-vivo* specimen.^20^^–^^25^^,^^27^^,^^31^^,^^32^^,^^37^ Of those, three used the bisected specimen to calculate the value of the signal.^22^^–^^24^ In addition, two studies compared a matched number of pixels from tumor and background in both *ex-vivo* and histopathology specimens for two timings.^14^^,^^28^ However, none of the studies went into sufficient detail regarding the way in which tumor or healthy tissue was marked, but stressed that the findings were confirmed on histopathology with fluorescence. Only Koch et al. calculated TBR relative to each patient.^20^

There were three studies in which the signal-to-background ratio was measured.^27^^,^^28^^,^^41^ Pop et al.^41^ assessed the area suspicious for tumor intraoperatively, Keating et al.^27^ examined the resection cavity, and Leiloglou et al.^28^ compared the difference between freshly excised tissue and histopathology specimen (which had undergone formalin fixation).^27^^,^^28^^,^^41^

A further four studies performed a qualitative analysis.^21^^,^^37^^,^^41^^,^^43^ Both Pop et al.^41^ and Smith et al.^23^ examined the cavity, whereas Koller et al.^21^ looked at both *in-vivo* and *ex-vivo* tissues. Conversely, Veys et al.^43^ compared benign and malignant lesions.

### 3.5 Image Processing

Four studies assessed accuracy using texture metrics.^14^^,^^20^^,^^28^^,^^33^ Leiloglou et al.,^28^ Leiloglou et al.,^33^ and Kedrzycki et al.^14^ performed image analysis via Fourier transformation for slope and intercept. Koch et al.^20^ capitalized on fSTREAM to streamline the intensity and spatial correlation.

Three studies mentioned the software used to determine TBR.^20^^,^^27^^,^^37^ This includes the HeatMap plugin within ImageJ (National Institutes of Health, Bethesda, Maryland),^27^ fSTREAM,^20^ and IC-Calc 2.0.^37^

### 3.6 Intraoperative FGS

Six studies utilized FGS intraoperatively.^24^^,^^30^^,^^31^^,^^34^^,^^35^^,^^40^ Three employed intralesional ICG which was used for both *in-vivo* guidance and assessing the cavity to confirm adequacy of resection. ^30^^,^^34^^,^^35^ Two used the PDE system (5.4% and 12.5% PMR, respectively),^30^^,^^35^ and one used the visual navigator system (10.5% PMR).^34^ The remaining three studies that reported PMR used conventional techniques (such as guidewires or seeds), thus PMR and reoperation rate were irrelevant to FGS.^24^^,^^31^^,^^40^ Of these, two provided *in-vivo* cavity images and *ex-vivo* images of BCS specimens, but surgical guidance was discretionary.^31^^,^^40^ Lastly, although marker localization was used intraoperatively, Smith et al. opted for additional cavity shaves in the event of fluorescence, resulting in a PMR of 17.8% and a reoperation rate of 8.9% with the LUM system.^24^

### 3.7 Diagnostic Accuracy

Seven studies assessed diagnostic accuracy using passive nonspecific fluorophores. Kedrzycki et al. observed a sensitivity of 69% and specificity of 97% in a study utilizing a custom built camera with pixel based processing to detect ICG fluorescence.^14^ However, when Leiloglou et al. applied texture metrics, a sensitivity of 75% and specificity of 89% were achieved.^28^ Liu et al. used the PDE to detect ICG signal and achieved a PPV and FPV of 100% and 0%.^35^ Veys et al. also assessed ICG by deploying the Fluobeam 800 imaging system and obtained a sensitivity and specificity of 94% and 32%, respectively.^37^ However, Pop et al. achieved a specificity of 60% and a PPV of 29% with the same combination.^41^ Zhang et al. developed their custom-built imaging system for MB signal detection and achieved a sensitivity and PPV of 63% and 79%, respectively.^32^ Lastly, Ottolino-Perry et al. utilized PRODIGI to detect 5-ALA’s metabolite (PpIX) signal and compared the diagnostic accuracy between the low dose (15  mg/kg) and high dose (30  mg/kg) cohorts.^22^ In the low-dose cohort, they presented a sensitivity, specificity, and PPV of 65%, 85%, and 77%, respectively.^22^ In the high dose cohort, they recorded a sensitivity, specificity, and PPV of 68%, 80%, and 75%, respectively.^22^

There were only two studies that reported the diagnostic accuracy of targeted fluorophores. Specifically, Smith et al. deployed the LUM imaging system for LUM015 detection and achieved 84% sensitivity and 73% specificity.^24^ Koch et al. achieved the highest sensitivity and specificity while capitalizing on the combination of bevacizumab–IRDye800CW and a custom-built imaging system, attaining 98% and 79%, respectively.^20^ The calculations for diagnostic accuracy can be found in the Supplementary Material.

### 3.8 Reproducibility

Fourteen studies provided inclusion/exclusion criteria,^13^^,^^14^^,^^20^^,^^22^^–^^25^^,^^27^^,^^28^^,^^30^^,^^31^^,^^37^^,^^38^ of which only Ottolino-Perry et al.^22^ had listed a minimum tumor size threshold. Six provided control samples; however, none of the studies included benign tumors for comparison.^14^^,^^22^^,^^23^^,^^28^^,^^30^^,^^34^ Thirteen studies described how TBR was calculated.^13^^,^^14^^,^^19^^–^^25^^,^^27^^,^^28^^,^^31^^,^^37^ Four studies provided a minimum TBR,^13^^,^^27^^,^^37^^,^^41^ with a further two using patient-normalized thresholds.^21^^,^^24^ Five compared individual cancer types,^14^^,^^19^^,^^28^^,^^30^^,^^37^ and five were able to detect DCIS.^13^^,^^19^^,^^23^^,^^31^^,^^36^ Three described the seniority of surgeons included in their study^14^^,^^28^^,^^30^ but no study described whether they were trained in FGS (Table 3).

**Table 3:** Assessment of consistency between studies. TBR, tumor-to-background ratio; DCIS, ductal carcinoma in-situ; FGS, fluorescence guided surgery.

Author/Ref. no.	Inclusion/exclusion criteria	Control provided	Benign tumors included	Minimum tumor size specified	TBR threshold	Pixel based processing	Individual cancer types compared	DCIS detectable	Level of surgeon included	Surgeons trained in FGS
Dintzis et al. ^19^	X	X	X	X	X	X	✓	✓	X	X
Keating et al. ^27^	✓	X	X	X	1.5	X	X	X	X	X
Kedrzycki et al. ^14^	✓	✓	X	X	X	✓	✓	X	✓	X
Koch et al. ^20^	X	X	X	X	✓*	✓	X	X	X	X
Koller et al. ^21^	X	X	X	X	X	X	X	X	X	X
Lamberts et al.^40^	✓	X	X	X	X	X	X	X	X	X
Lee et al.^34^	X	✓	X	X	X	X	X	X	X	X
Leiloglou et al. ^33^	X	X	X	X	X	✓	X	X	X	X
Leiloglou et al.^28^	✓	✓	X	X	X	✓	✓	X	✓	X
Liu et al.^35^	X	X	X	X	X	X	X	X	X	X
Ottolino-Perry et al.^22^	✓	X	X	X	1.3 (but only in histopathology)	X	X	X	X	X
Park et al.^36^	✓	✓	X	✓	X	X	X	X	X	X
Pop et al.^41^	X	X	X	X	X	X	X	✓	X	X
Smith et al.^23^	✓	✓	X	X	X	✓	X	✓	X	X
Smith et al.^24^	✓	X	X	X	✓*	✓	X	X	X	X
Tong et al.^30^	✓	✓	X	X	X	X	✓	X	✓	X
Tummers et al.^31^	✓	X	X	X	X	X	X	✓	X	X
Tummers et al.^38^	✓	X	X	X	X	X	X	X	X	X
Unkart et al. ^25^	✓	X	X	X	X	X	X	X	X	X
Veys et al.^37^	✓	X	X	X	1.3	X	✓	X	X	X
Zhang et al.^32^	✓	X	X	X	1.3	X	X	✓	X	X

✓* = TBR threshold calculated per patient.

### 3.9 Adverse Events

There were no serious adverse events relating to any of the fluorescence imaging systems. Only one patient had a hematoma attributed to the device (due to pressure applied in the cavity); however, this resolved spontaneously.^24^

Eight studies reported adverse events due to drug-related side effects.^21^^–^^24^^,^^27^^,^^31^^,^^32^^,^^38^ These included: one patient with mild nausea successfully treated with IV diphenhydramine,^27^ another patient with untreated nausea and one with hot flushes (both of which recovered spontaneously),^21^ five patients with mild transient pain on injection of MB (three of which were successfully treated with saline flush),^31^^,^^32^ one had blue skin discoloration after extravasation of LUM015 (which resolved within 3 months),^24^ and one with self-limiting hypersensitivity to EC17 (abdominal discomfort, itching throat, sneezing) during injection.^38^ Furthermore, there was one case of sunburn with ALA; however, it was due to a patient not abiding by the post-operative protocol.^22^

One patient experienced adverse events related to the anesthetic, exhibiting transient hypertension on induction and awakening.^23^ Furthermore, there was a case of transient peri-operative hypertension and another case with peri-operative nausea; however, both were reported to be unlikely related to the trial.^24^

## 4 Discussion and Conclusions

There is an overwhelming need to improve precision during BCS,^10^^,^^44^^–^^47^ but current localizing techniques are unable to provide surgeons with sufficient information to guarantee entire tumor removal.^12^ Eliminating the need for a second surgery would benefit patients, alleviating psychological stress, reducing complications, and improving cosmetic outcomes and quality of life. In addition, the hospital would benefit from decreased use of resources, improved workflow, and by negating the costs of a re-operation.^48^ The combination of these factors has led to substantial research interest in FGS.

In this review, there were only three studies wherein resection was guided by ICG fluorescence, all of which were with intralesional injection.^30^^,^^34^^,^^35^ As the remaining trials utilized conventional techniques (e.g., wires, seeds), the results of these trials arguably reflect the radiologist’s competencies, rather than FGS.

It was interesting to note that there was no significant difference in TBR between patients who had received NACT versus those who had undergone primary surgery.^32^ One may have expected a lower TBR given fibrosis after NACT due to the dense tissue possibly preventing fluorophore passively leaking. Perhaps this is compensated for by the increased reflection of fibrotic tissue. However, for the purpose of cosmesis, it is critically important to differentiate between tumor and fibrosis in order to optimize the volume of tissue resected.

The two studies by Kedrzycki et al.^14^ and Leiloglou et al.^28^ analyzed different administration timings of ICG. The higher signal in the angiography cohort may be attributed to the increased concentration of the ICG in the blood vessels as the TBR is captured prior to the excretion of ICG. Alternately, the EPR timing occurs after ICG washout and visualizes the tumor only having a fraction of the ICG present.

One study compared TBR of different tumor grades and observed grades 2 and 3 had a greater TBR than grade 1 cancers.^20^ Only one study compared tumor histological subtypes and observed no statistically significant difference.^14^ This is surprising as one could have expected IDC to provide a stronger signal than invasive mucinous carcinoma, due to IDC’s increased vascularity and density of tissue.

Furthermore, given that the TBR threshold considered sufficient for *in-vivo* studies is >1.5,^49^ there was only one study which provided the minimum clinically relevant contrast.^27^ However, the two trials that set lower TBR parameters for success were still able to meet the recommended threshold.^32^^,^^37^ The remaining studies which did not set any threshold were also able to surpass the minimum TBR.^14^^,^^20^^,^^23^^,^^25^^,^^28^^,^^31^^,^^38^

In addition, only Ottolino-Perry et al. specified a minimum 2 cm threshold for tumor size as part of their inclusion criteria.^22^ This minimum size limitation may have been implemented in view of the camera’s intrinsic limitation of working distance and field of view for detecting smaller tumors. Therefore, a large minimum size threshold is a severe limitation as it does not address small tumors or DCIS (which is the leading cause of positive margins).^7^ Since DCIS is micrometers in size, an imaging device would ideally be able to accommodate DCIS imaging by incorporating the appropriate lens system. However, the combination of lenses for microscopy with commercially available camera sensor resolution would only allow for a very small field of view to be inspected at a time. Therefore, the technique would not be well suited to real-time surgery where scanning the entire surgical field would be cumbersome. Alternatively, excised tissue margins could be inspected intraoperatively with the microscopy mode. However, given the weak DCIS fluorescence signal, camera sensors would have to be highly sensitive to fluorescence photons (known as camera quantum efficiency), to successfully capture the image.

None of the studies describe whether there was any previous training for surgeons in FGS or how many attempts it took to overcome the learning curve, with only one reporting the surgeon’s seniority.^28^ Such details are crucial to future trials to assess how much training is required, and whether surgical expertise impacts on signal quality and diagnostic accuracy. These two factors will help determine how many attempts are needed and by what level of surgeon before FGS can be applied *in-vivo* with sufficient accuracy.

None of the camera systems using targeted versus EPR approaches were able to surpass the minimal accuracy for clinical adoption. The studies that came closest were the study by Koller et al. which used the SurgVision camera system in combination with the targeting bevacuzimab800 and achieved a sensitivity of 88% and a sensitivity of 89%.^21^ Alternately, in our studies, we used our in-house camera in combination with passive ICG and achieved a sensitivity of 69% and specificity of 72%.^14^^,^^28^

It is impossible to determine the superiority of any one camera system given methodological heterogeneity in trials. The only way to compare camera systems would be to hold constant other important experimental factors in the protocol, such as fluorophore type, dose and timing of administration, camera settings for data collection, and reporting structure. Furthermore, it would be worthwhile to compare the breast cancer subtypes (as the majority are IDC), immunophenotypes (ER/PR/Her2 status), as well as any pre-treated cases (NACT or hormonal therapy). The subtypes would be particularly important in the case of DCIS, which accounts for the majority of PMR cases.^7^ It would also be valuable to include benign disease, such a fibrocystic change and cellular atypia (e.g., flat epithelial atypia and atypical intraduct proliferations) as these may result in false positives. In addition, these studies should also report on ergonomics, such as camera useability, distance from camera to surface, and the corresponding field of view. Toward methodological consistency and consistency in reporting, we propose a checklist of details that future studies include in order to facilitate comparison between FGS camera systems for BCS (Table 4).

**Table 4:** Checklist for future camera studies.

Checklist	Y/N
Inclusion and exclusion criteria	—
Camera system specifications (e.g., name, manufacturer, spectrum of wavelengths captured, ergonomics)	—
Trial registry number	—
Trial methodology (e.g., prospective, randomized, blinded, etc.)	—
Powered study population (to determine whether a statistically significant difference between tumor and healthy tissue exists)^a^	—
Exogenous contrast agent being used (i.e., name, dose, route, and timing of administration, target)	—
Studies should acquire all images (e.g., tumor *in situ*, cavity, specimen, histopathology cut-up, etc.)	—
Patient demographics (e.g., height, weight, BMI, etc.)	—
Clinicopathologic data (e.g., non-invasive/invasive, size, histological subtype, immunophenotype, etc.)	—
Complications	—
TBR calculation (e.g., qualitative or quantitative analysis, areas being marked, processing, and software used)	—
Diagnostic accuracy (e.g., sensitivity, specificity, PPV, NPV, etc.)	—
The seniority of the surgeons utilizing FGS and if any training was provided prior to using FGS	—

TBR, tumor background ratio; PPV, positive predictive value; NPV, negative predictive value; FGS, fluorescence guided surgery.

Once FGS is the sole technology being used intraoperatively, studies should report the number of patients with positive margins, reoperations, and disease recurrence. In addition, once the data above are available, then a cost-analysis comparing the gold standard to FGS should be performed.

a If there is any comparison within study (i.e., timings), the study would require repeating the power calculation.

In conclusion, the translation of these camera systems to be used in breast cancer remains in its early stages, as the majority of systems are either under development or still being assessed in prospective trials (NCT04815083). Therefore, although FGS in breast cancer shows great promise, further clinical trials are required prior to clinical adaptation. It is only once the limitations are addressed that diagnostic accuracy can be useful in distinguishing between camera systems.

## 5 Appendix: Search Strategy

Embase, MEDLINE, Web of science, and Scopus were systematically searched for all articles published before April 2022. The search was conducted using the following Medical Subject Headings (MeSH) terms in conjunction (and/or) with operators: (‘fluorescence imaging’[All Fields] OR ‘near infrared fluorescence’[All Fields] OR ‘near infrared’[All Fields] OR ‘NIRF’[All Fields] OR ‘NIR’[All Fields] OR ‘IRF’[All Fields’ OR ’infrared fluorescence’[All Fields] OR ‘fluorescence’[MeSH Terms]) AND (‘breast cancer*’[All Fields] OR ‘breast tumo?r’[All Fields] OR ‘breast maligananc*’[All Fields] OR ‘breast neoplasm*’[All Fields] OR ‘breast cancer’[MeSH Terms]) AND (‘intraoperative’[All Fields] OR ‘intra-operative’[All Fields] OR ‘breast surger*’[All Fields] ‘breast conserving surger*’[All Fields] OR ‘surgery’[MeSH Terms]) AND (‘fluorescence guided surger*’[All Fields] OR ‘FGS’[All Fields] OR ‘fluorescence imaging’[All Fields] OR ‘fluorescence imaging system’[All Fields] OR ‘imaging system’[All Fields] OR ‘fluorescence planar imaging’[All Fields] OR ‘fluorescence imaging’[MeSH Terms]). For different databases, the search terms were edited and updated as required. Additional reports were identified using the CLEARER database and Google Scholar through citation tracking.

## Supplementary Material



## Data Availability

Data sharing is not applicable to this article, as no new data were created or analyzed.
