# Supplementary material for: Fluorescence guided surgery imaging systems for breast cancer identification: a systematic review
Source: J Biomed Opt. 2024 Mar 4;29(3):030901. doi: 10.1117/1.JBO.29.3.030901 (PMC10911048; doi:10.1117/1.JBO.29.3.030901)
Supplement: Supplementary file 1 [file JBO_029_030901_SD001.docx]

**Calculations**

Tumor background ratio (TBR)= tumor fluorescence/ background fluorescence.

Diagnostic accuracy calculations:

Sensitivity = true tumor / (true tumor + false healthy)

Specificity = false tumor / (false tumor + true healthy)

Positive predictive value = true healthy / (true healthy + false healthy)

Negative predictive value = false tumor / (false tumor + true tumor)

Accuracy = (true tumor + true healthy) / (true tumor + false tumor + true healthy + false healthy)
